# Human metabolic response to systemic inflammation: assessment of the concordance between experimental endotoxemia and clinical cases of sepsis/SIRS

**DOI:** 10.1186/s13054-015-0783-2

**Published:** 2015-03-03

**Authors:** Kubra Kamisoglu, Beatrice Haimovich, Steve E Calvano, Susette M Coyle, Siobhan A Corbett, Raymond J Langley, Stephen F Kingsmore, Ioannis P Androulakis

**Affiliations:** Department of Chemical and Biochemical Engineering, Rutgers University, Piscataway, NJ 08854 USA; Department of Surgery, Rutgers - Robert Wood Johnson Medical School, New Brunswick, NJ 08901 USA; Department of Respiratory Immunology, Lovelace Respiratory Research Institute, Albuquerque, NM 87108 USA; Center for Pediatric Genomic Medicine, Children’s Mercy, Kansas City, MO 64108 USA; Departments of Pediatrics and Obstetrics/Gynecology, University of Missouri, Kansas City, MO 64108 USA; Department of Biomedical Engineering, Rutgers University, 599 Taylor Road, Piscataway, NJ 08854 USA

## Abstract

**Introduction:**

Two recent, independent, studies conducted novel metabolomics analyses relevant to human sepsis progression; one was a human model of endotoxin (lipopolysaccharide (LPS)) challenge (experimental endotoxemia) and the other was community acquired pneumonia and sepsis outcome diagnostic study (CAPSOD). The purpose of the present study was to assess the concordance of metabolic responses to LPS and community-acquired sepsis.

**Methods:**

We tested the hypothesis that the patterns of metabolic response elicited by endotoxin would agree with those in clinical sepsis. Alterations in the plasma metabolome of the subjects challenged with LPS were compared with those of sepsis patients who had been stratified into two groups: sepsis patients with confirmed infection and non-infected patients who exhibited systemic inflammatory response syndrome (SIRS) criteria. Common metabolites between endotoxemia and both these groups were individually identified, together with their direction of change and functional classifications.

**Results:**

Response to endotoxemia at the metabolome level elicited characteristics that agree well with those observed in sepsis patients despite the high degree of variability in the response of these patients. Moreover, some distinct features of SIRS have been identified. Upon stratification of sepsis patients based on 28-day survival, the direction of change in 21 of 23 metabolites was the same in endotoxemia and sepsis survival groups.

**Conclusions:**

The observed concordance in plasma metabolomes of LPS-treated subjects and sepsis survivors strengthens the relevance of endotoxemia to clinical research as a physiological model of community-acquired sepsis, and gives valuable insights into the metabolic changes that constitute a homeostatic response. Furthermore, recapitulation of metabolic differences between sepsis non-survivors and survivors in LPS-treated subjects can enable further research on the development and assessment of rational clinical therapies to prevent sepsis mortality. Compared with earlier studies which focused exclusively on comparing transcriptional dynamics, the distinct metabolomic responses to systemic inflammation with or without confirmed infection, suggest that the metabolome is much better at differentiating these pathophysiologies. Finally, the metabolic changes in the recovering patients shift towards the LPS-induced response pattern strengthening the notion that the metabolic, as well as transcriptional responses, characteristic to the endotoxemia model represent necessary and “healthy” responses to infectious stimuli.

**Electronic supplementary material:**

The online version of this article (doi:10.1186/s13054-015-0783-2) contains supplementary material, which is available to authorized users.

## Introduction

Sepsis is defined as the combination of an infection with multiple features of ‘systemic inflammatory response syndrome’ (SIRS) [1] and is one of the oldest and most enigmatic conditions in medicine. There are more than a million cases of sepsis per year in the United States [2] and it is estimated that there are 19 million cases per year worldwide [3,4]. According to the Centers for Disease Control, the cost of hospitalization is in the order of $15 billion, with an anticipated further increase in the future [5]. Despite several decades of intensive research and efforts to bring new therapies to the bedside, the number of cases and sepsis-associated deaths are still soaring [3,6]. Current treatment guidelines include cardiorespiratory resuscitation and non-specific protocols aimed at mitigating immediate threats of uncontrolled infection [3]. A significant barrier to progress is the perceived inadequacy of experimental models that can reproduce the pathophysiology of the disease in humans.

The high degree of variability among patients and multiple aspects of the disease, including patient gender, age and comorbidities complicate the design of relevant experimental models and clinical studies. Moreover, the initiating cause of infection and the physiologic responses that follow are also highly variable [7]. All these factors explain, at least in part, the difficulty in translating experimental results to the clinic and, consequently, the lack of success in the development of effective therapies [8].

Endotoxemia, an experimental model in which healthy volunteers are intravenously administered a form of endotoxin (lipopolysaccharide, LPS, a major component of Gram-negative bacteria outer membrane and a Toll-like receptor 4 (TLR4) agonist) [9], has served as a valuable experimental venue for more than six decades [10-12]. It is a model of systemic inflammation, rather than a true mimic of sepsis. Nonetheless, early transient physiochemical changes and biochemical pathway activation in this model are strikingly similar to those observed during the early hyperdynamic phase of resuscitated injury and infection [13]. The LPS challenge triggers chills, myalgias, nausea, and an increase in core body temperature and heart rate, most of which begin to abate within six to eight hours [11,13,14]. Genome-wide analyses of circulating leukocytes revealed transcriptional signatures indicative of changes in protein translation and glycolysis [15], which shared similar characteristics with those observed in trauma patients [16]. These studies helped elucidate the intricate regulatory schemes governing the response to endotoxemia [16,17] and provided the foundations for *in silico* models of systemic inflammation [18-24]. More recently, we documented the effects of LPS-induced inflammation on the whole body metabolism in humans [25]. In contrast with other methods applied to the endotoxemia model, metabolomics reflects the combined output of all tissues in the body [26]. In that study [25], plasma samples, collected from healthy subjects during 24-hours post challenge with LPS, were subjected to non-targeted biochemical profiling, revealing the temporal changes in the plasma metabolome. Unsupervised multivariate analyses identified prominent changes in lipid and protein metabolism, which peaked at six hours post LPS infusion. Subsequently, to understand better how the inflammatory responses at the level of cells and whole body correlate in humans, we integrated the analysis of the plasma metabolome with that of the leukocyte transcriptome [27].

In [28], an integrated analysis of clinical features, plasma metabolome and proteome described the pattern of metabolic perturbations in critically ill patients presenting with symptoms of SIRS or sepsis. This study, the first of its kind, examined clinical features as well as the plasma metabolome, and proteome, of patients upon arrival at the emergency department (ED) and 24 hours later. An important and novel outcome of the study was the realization that metabolic differences could ultimately be used as markers predicting survival.

Since the endotoxemia model utilizes LPS, rather than intact bacteria, there is an ongoing concern that data derived from this model are of limited relevance to our understanding of sepsis-induced inflammatory mechanisms, although recent analyses of the leukocyte transcriptome seemed to argue otherwise [15]. The availability of new metabolomic data [25,28] offered the opportunity to compare responses detected in LPS-challenged subjects to those of critically ill patients at the level of the entire organism. In this retrospective study we aimed to objectively determine the relevance of the information content gained by parallel analyses of LPS-challenged subjects [25] and patients with or without community-acquired sepsis [28]. Our study identified a core response that was in agreement with what was observed in sepsis patients. Response to systemic inflammation without apparent infectious stimuli such as what is observed in SIRS was shown to have distinct features that may make it uniquely recognizable at the metabolomics level. Metabolic changes in the patients who are recovering shifted towards an endotoxemia response pattern, strengthening the idea that the endotoxemia model represents necessary and ‘healthy’ responses to an infectious stimulus.

## Material and methods

### Metabolic data

**Figure 1:**
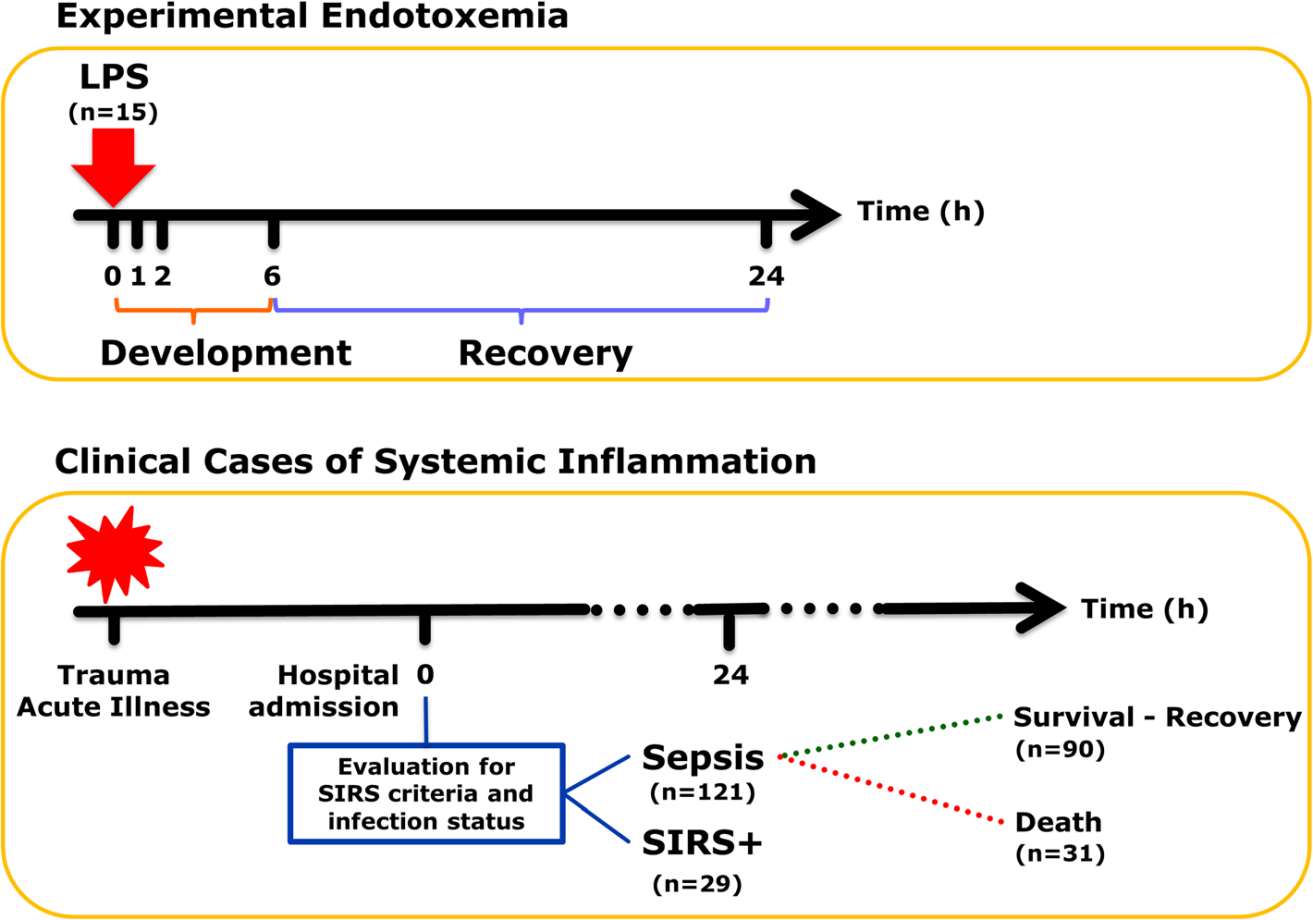
**Schematic description of the experimental and clinical sources of data used.**

This is a retrospective analysis utilizing metabolomes obtained from subjects who participated in an experimental endotoxemia study and from patients with or without community-acquired sepsis. In brief, as previously described [25], healthy volunteers participated in an endotoxemia study, after providing written, informed consent under guidelines approved by the Institutional Review Board of Rutgers - Robert Wood Johnson Medical School. Inclusion criteria were age between 18 to 40 years, and normal general health as demonstrated by medical history and physical examination as well as laboratory testing. National Institutes of Health (NIH) Clinical Center Reference Endotoxin at a bolus dose of 2 ng/kg body weight was administered to 15 subjects. Blood samples were collected at t = 0, prior to treatment, and at 1, 2, 6, and 24 hours post-treatment (Figure 1, top). Mass spectrometry (MS) based biochemical analysis of the plasma samples was performed by Metabolon (Durham, NC, USA) according to previously published methods [29]. All details of the study design and biochemical analysis are available in [25].

Metabolomic data for the clinical cases of systemic inflammation were obtained from the Community Acquired Pneumonia and Sepsis Outcome and Diagnostics (CAPSOD) study [28]. Approval for this study was obtained by institutional ethics committees and details were filed at ClinicalTrials.gov (NCT00258869). Protocols and identified clinical features in the different classes of patients were previously published. The study [28] included 1,152 individuals with suspected, community-acquired sepsis (acute infection and ≥2 SIRS criteria) in the emergency departments at three urban, tertiary-care hospitals in the United States between 2005 and 2009. Each patient or their legal designates provided informed consent. Medical history, physical examination, and acute illness scores (Acute Physiology and Chronic Health Evaluation II (APACHE II) and Sequential Organ Failure Assessment (SOFA)) were recorded at enrollment (t_0,clinical_) and 24 hours later (t_24,clinical_). Infection status and outcome through day 28 were independently determined by board-certified clinicians. Clinical care given for the patients was not standardized and was determined by individual providers. After independent audit of infection status and outcomes, 150 patients were chosen for derivation studies. Non-targeted mass spectrometry based analyses of the patients’ blood samples were done by Metabolon similarly to the endotoxemia study.

The 150 patients chosen for derivation studies within the CAPSOD cohort were classified to represent cases of uncomplicated sepsis (n = 27), severe sepsis (n = 25), septic shock (n = 38), non-infected SIRS (‘ill’ controls, indicated as SIRS, presumed septic at enrollment but later determined to have noninfectious reasons for SIRS; n = 29) or sepsis non-survival (SNS, n = 31) (Figure 1, bottom). No significant differences among subgroups of sepsis survivors (uncomplicated sepsis, severe sepsis, septic shock) were reported for plasma metabolites [28]. Therefore, the data from these patients were collapsed into a single group referred to as sepsis survivors (SS, n = 90) for the purposes of this study. Furthermore, in the first part of the analysis, metabolic data from SS and sepsis non-survivors (SNS) were pooled to assess the similarities and differences between sepsis and non-infected SIRS, and referred to as the Sepsis group (n = 121). In the subsequent analysis, data from the SS and SNS groups were used individually to investigate the association of metabolic changes in endotoxemia with those observed in either surviving or non-surviving sepsis patients.

### Data analysis

MS analysis of plasma samples from the human endotoxemia study provided temporal information on 366 metabolites at five time points. Previous results of the principal component analysis on this dataset showed that the six hour time point (t_6_) was the most critical since the maximum difference between control and treatment groups was observed at this time point [25]. This agreed well with prior transcriptional studies indicating that the maximal change in leukocyte gene expression was observed six hours after the LPS administration [16,30]. Therefore, this data point was considered to represent the peak of metabolic response to endotoxemia and used as reference for the assessment of concordance between experimental and clinical data in this study. MS analysis of the samples from the CAPSOD study, on the other hand, had identified 370 metabolites at t_0,clinical_ (time of hospital admission) and 401 metabolites at t_24,clinical_ (24 hours after admission). In this study, both clinical and experimental datasets were individually normalized by setting the median equal to 1. Missing values were imputed with the observed minimums after normalization. The metabolite lists were consolidated. Only the metabolites commonly identified in the endotoxemia [25] and clinical [28] studies were analyzed further. The final dataset included 177 common metabolites from both studies [25,28]. Outliers were removed using the median absolute deviation, MAD = 1.4826 × |(x_i_ – Median_j_(x_j_)|, of each metabolite, determined in each individual group [31,32]. Subsequently, the score for each data point was calculated ${\mathrm{z}}_{\mathrm{i}} = \frac{\left|{\mathrm{x}}_{\mathrm{i}}-{\mathrm{Median}}_{\mathrm{j}}\left({\mathrm{x}}_{\mathrm{j}}\right)\right|}{\mathrm{MAD}}$ and data points with a score above 3 were removed from the dataset. The number of removed outliers for each group is reported in Additional file 1: Table S1.

**Figure 2:**
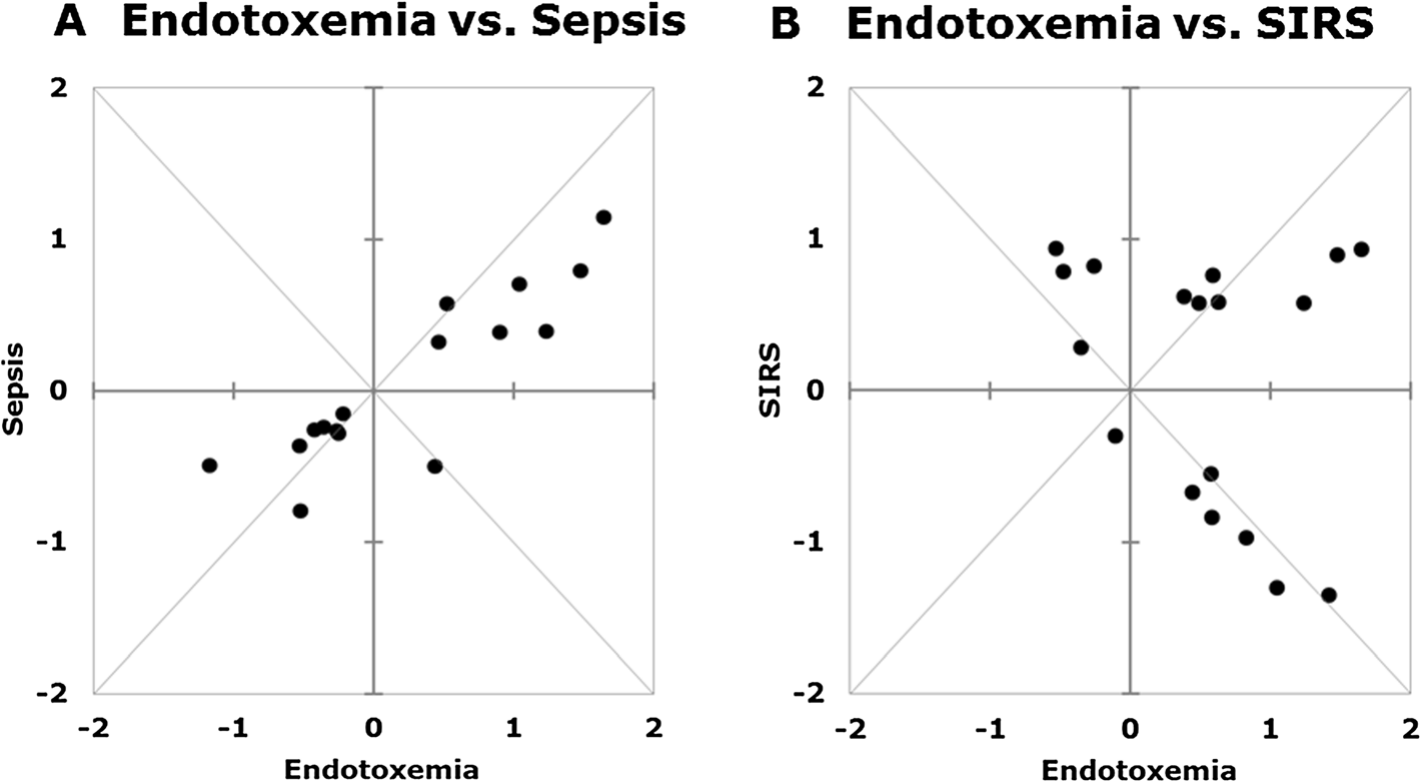
**Scatter plots show the direction and extent of changes in the metabolites that significantly deviated from baseline in sepsis (A) and SIRS (B) groups in relation to corresponding trends in endotoxemia.** For the clinical data, plots reflect the maximum observed change from the baseline if that particular metabolite was found to be significant in both time points, t_0,clinical_ and t_24,clinical_. SIRS, severe inflammatory response syndrome.

The baseline of the human endotoxemia studies, that is, samples collected before LPS administration (t_0,LPS_), defined the ‘baseline’ in this study. We identified the six hour time point as the peak of the metabolic response in the endotoxemia model in our previous metabolomics study [25], as well as transcriptomic analysis [17,18], and hypothesized that this time point represents the point of transition from the development and recovery phases of the response. For the clinical data collection, the starting point was the time of hospital admission (t_0,clinical_), whereas the second clinical time point was 24 hours later (t_24,clinical_). Since the data obtained for the clinical patients lack internal controls, for obvious reasons, the responses of each group of patients, as well as the endotoxemia subjects, were compared independently to the healthy baseline (t_0,LPS_). For comparing the means of metabolites in each condition relative to the healthy baseline, Welch’s t-test was used without assuming equal variances (α = 0.05). The number of subjects in the experimental endotoxemia group (n = 15) was much smaller than the number of patients in the clinical groups to assume normal distribution required for the t-test. However, at both t_0,LPS_ and t_6,LPS_, the data passed the Kolmogorov-Smirnov test for each metabolite allowing the application of the t-test. Q-values were calculated according to the Benjamini and Hochberg procedure [33] and metabolites having a *P*- and a q-value less than 0.05 are called significant. We also evaluated how dispersed the data for each metabolite is in clinical cases with respect to those at the baseline. Variances of the significant metabolites in each condition were also plotted relative to the variances at the baseline and are shown in Additional file 2: Figure S1. The direction and magnitude of changes in plasma metabolite concentrations were determined based on log2 fold changes from t_0,LPS_ for both clinical groups and plotted against those observed for endotoxemia as shown in Figure 2. The full list of the metabolites, their significance in each condition, the direction and magnitude of the changes relative to the baseline is provided in Additional file 3: Table S2.

We lastly focused on changes in metabolites within subpopulations of sepsis patients who ultimately survived, and those who did not. For this purpose, dynamics within the sepsis group were examined using the data from the SS and SNS groups. Metabolites that statistically differed between these groups at either time point were determined by t-test as described earlier. The magnitude of changes in plasma metabolites relative to t_0,LPS_ were calculated for each group. These changes were compared between the SS and SNS groups at both time points and with the endotoxemia group at t_6,LPS._

## Results and discussion

Endotoxemia induced by elective administration of LPS to healthy subjects has served as an invaluable tool for obtaining mechanistic insight into homeostatic inflammatory responses. Previous studies compared transcript- and protein-expression patterns in immune cells obtained from LPS treated subjects and trauma patients, revealing significant overlap [15,34]. More recently, metabolomics analyses in both LPS-administered subjects [25] and patients with symptoms of systemic inflammation at time of presentation to emergency departments were published [28,35]. Building on these prior studies [26,29], here we aimed to objectively compare metabolic indices obtained from experimental studies and clinical sources.

The inherent dynamics of a clinical and an experimental study are obviously disparate although they focus on related physiologic phenomena. The first time point in a clinical study is generally at a point that had already deviated from what can be called a ‘healthy state,’ whereas experimental studies usually measure divergence from the ‘healthy state’ under controlled conditions. In this study, we aimed to evaluate the significance of observed metabolic perturbations in endotoxemia and how they relate to corresponding changes observed in patients with symptoms of community-acquired sepsis at the time of presentation to the emergency departments. In line with our objective, we chose to evaluate each condition and each time point based on its deviation from one common baseline that reflects a healthy state (t_0,LPS_). Table 1 shows the number of metabolites that had a significantly different concentration compared to t_0,LPS_ at each time point available for each condition. The first column in Table 1, shows the total information content of the final consolidated dataset with total number of metabolites associated with each metabolic super-pathway. The complete list of metabolites, their pathway classification, and extent of changes from baseline are provided in Additional file 3: Table S2. At the peak of the response to LPS, that is, at t_6,LPS_, there were 83 metabolites (47% of the total 177 common metabolites) which significantly deviated from baseline. In contrast, the number of metabolites that significantly differed from baseline for the clinical groups was considerably smaller (varying between 19 to 26 metabolites, or 11% to 15% of the total 177 common metabolites). We hypothesize that the much larger number of metabolites that changed significantly in response to endotoxin as compared to the clinical cases reflects, at least in part, the fundamental difference between physiologic variability of responses elicited in subjects who participated in the controlled endotoxemia study and patients. The endotoxemia study cohort included relatively young and healthy subjects whereas the patient cohort that participated in the CAPSOD study was variable, in terms of age and comorbidities among others. In addition, the trigger itself, that is, LPS, activates a single TLR4-dependent signaling pathway, whereas infectious agents and trauma activate multiple ones, leading to greater variability in responses. In order to evaluate the level of dispersion in the clinical data with respect to the experimental, the variance of each significant metabolite at each clinical condition was calculated and plotted against the corresponding variance at the baseline. These plots are show in Additional file 2: Figure S1. As highlighted in these plots, variances of the metabolites measured in the patients were statistically higher than those measured in the endotoxemia study participants at the baseline, t_0,LPS_, reflecting the fundamental differences in variability between the two groups.

**Table 1:** **Number of significantly changed metabolites and metabolic super-pathways that they belong to, determined for LPS-challenged subjects and patient groups**

		**LPS (number = 15)**	**Sepsis (number = 121)**		**SIRS (number = 29)**	
**Super pathway**	**Total**	**t6**	**t0**	**t24**	**t0**	**t24**
Amino acid	55	28	3	5	3	4
Carbohydrate	16	4	1	2	2	2
Cofactors and vitamins	7	3	2	1	2	1
Lipid	75	36	10	16	12	16
Energy	4	4	0	1	1	1
Nucleotide	8	3	0	0	1	0
Peptide	3	3	0	0	0	0
Xenobiotics	9	2	3	1	0	1
**Total**	**177**	**83**	**19**	**26**	**21**	**25**

Significance was determined by comparing responses of each group of patients, as well as endotoxemia subjects, to the healthy baseline (t_0,LPS_) individually. Welch’s t-test was used and with correction for multiple comparisons by the Benjamini and Hochberg procedure. (α = 0.05). Metabolites having a *P*- and a q-value less than 0.05 are called significant. (The complete list is available in Additional file 3: Table S2). LPS, lipopolysaccharide; SIRS, severe inflammatory response syndrome.

Next, we sought to determine the similarities and differences among the subsets of significant metabolites that changed significantly in the sepsis and SIRS groups which were also significant in endotoxemia. Our intention was to be maximally inclusive of the clinically observed changes. Therefore, we focused on metabolites that were significantly different from the baseline at either one of the two clinical time points as well as in endotoxemia. Table 2A lists the metabolites common to endotoxemia and sepsis and Table 2B lists those common to endotoxemia and SIRS. Metabolites that are common to both lists A and B are typed in bold. Triangles depict the direction (apex up: increase, apex down: decrease) and magnitude (one triangle: less than two fold change, two triangles: more than two fold change) of the difference relative to the baseline (t_0,LPS_). Although the total number of metabolites in common with endotoxemia is close for the two clinical cases (16 in Table 2A and 18 in Table 2B), the agreement between the directions of change is strikingly different. Bilirubin, docosapentaenoate (DPA) and palmitolate were the only three metabolites common to the LPS, sepsis and SIRS groups, which changed in the same direction. Of the 16 metabolites common to LPS and sepsis (Table 2A), 15 changed in the same direction. Only one, xylose, changed in an opposite direction. In marked contrast, of the total 18 metabolites common to the endotoxemia and SIRS groups, 10 changed in the opposite direction (Table 2B). Scatter plots shown in Figure 2A and B highlight this distinction in response. The axes of the scatter plots indicate the log2 fold changes in metabolite concentrations. The x-axes show the change at t_6,LPS_ from baseline, t_0,LPS_. The y-axes show the maximum change in the clinical data at either t_0,clinical_ or t_24,clinical_, relative to t_0,LPS_ (if the changes at both time points were significant, the higher of the two values is shown). Positive direction shows an increase in concentration, while negative shows a decrease. Accordingly, the concentrations of metabolites in the first and third quadrants change in parallel with the observations in endotoxemia; while the ones in the second and fourth quadrants change in the opposite direction. The response reflected by the direction and magnitude of change in septic patients agrees well with response to LPS within this common subset. However, for the SIRS group, the directions of change are not in agreement with those in endotoxemia for more than half of the metabolites. This suggests that, at the whole body metabolome level, SIRS elicits a unique response with distinctive features. One such feature is the marked decrease in sulfated androgenic hormones (epiandrosterone sulfate, androsterone sulfate, dehydroisoandrosterone sulfate (DHEA-S), 5alpha-pregnan-3beta,20alpha-diol disulfate) (Additional file 3: Table S2). Lower plasma concentration of one of these metabolites, DHEA-S, has previously been associated with other systemic inflammatory diseases, such as systemic lupus erythematosus and inflammatory bowel disease [36]. This supports the idea that the inflammatory response without apparent infectious stimuli might elicit distinctive features not shared with sepsis or endotoxemia. It has been previously suggested that acute inflammatory stresses from different etiologies result in highly similar responses in humans at the genomic level [37]. The observed distinct metabolomic responses to systemic inflammation with or without confirmed infection, however, suggest that the metabolome is much better at differentiating and understanding the various pathophysiologies of the different systemic inflammatory responses. Identified unique features of the inflammatory response in different contexts may aid in improving the diagnosis or the development of more targeted therapies.

**Table 2:** **Metabolites which are significantly different than the heathy baseline (t**_**0,LPS**_ **) in the experimental condition and either of the two time points in the clinical conditions**

**A**		**LPS**	**Sepsis**	
**Metabolite name**	**Super pathway**	**t6**	**t0**	**t24**
2-hydroxybutyrate (AHB)	Amino acid	▲	=	▲
mannose	Carbohydrate	▲	=	▲
**xylose**	Carbohydrate	▲	=	▽
hexanoylcarnitine (C6)	Lipid	▲	▲	▲
**bilirubin**	Cofactors and vitamins	▲▲	▲	▲▲
**docosapentaenoate (DPA; 22:5n3)**	Lipid	▲▲	=	▲
**palmitoleate (16:1n7)**	Lipid	▲▲	▲	▲
pregnen-diol disulfate	Lipid	▲▲	▲	▲
citrulline	Amino acid	▽	▽	▽
histidine	Amino acid	▽	=	▽
**serine**	Amino acid	▽	=	▽
threonine	Amino acid	▽	▽	=
2-palmitoyl-GPC (16:0)	Lipid	▽	▽	▽
uridine	Nucleotide	▽	=	▽
gamma-glutamyltyrosine	Peptide	▽	=	▽
catechol sulfate	Xenobiotics	▽▽	▽	=
**B**		**LPS**	**SIRS**	
**Metabolite name**	**Super pathway**	**t6**	**t0**	**t24**
alpha-ketobutyrate	Amino acid	▲	=	▽
N-acetylglycine	Amino acid	▲	=	▽
**xylose**	Carbohydrate	▲	=	▽
citrate	Energy	▲	▲	▲
arachidonate (20:4n6)	Lipid	▲	▲	▲
docosahexaenoate (DHA; 22:6n3)	Lipid	▲	▲	=
eicosapentaenoate (EPA; 20:5n3)	Lipid	▲	=	▲
octadecanedioate (C18)	Lipid	▲	=	▽
**bilirubin**	Cofactors and vitamins	▲▲	▲	=
3-hydroxybutyrate (BHBA)	Lipid	▲▲	=	▽▽
**docosapentaenoate (DPA; 22:5n3)**	Lipid	▲▲	▲	=
hexadecanedioate (C16)	Lipid	▲▲	▽▽	▽
**palmitoleate (16:1n7)**	Lipid	▲▲	▲	▲
5-oxoproline	Amino acid	▽	▽	=
proline	Amino acid	▽	=	▲
**serine**	Amino acid	▽	▲	=
1-linoleoyl-GPC (18:2)	Lipid	▽	▲	▲
1-oleoyl-GPC (18:1)	Lipid	▽	=	▲

**A** lists the metabolites common for endotoxemia and sepsis; **B** lists those common for endotoxemia and SIRS. (=: no significant difference from t_0,LPS_. ▲/▽: less than two fold difference from t_0,LPS_; ▲▲/▽▽: more than two fold difference from t_0,LPS_; metabolite name in bold: common to both lists A and B). LPS, lipopolysaccharide; SIRS, severe inflammatory response syndrome.

Next we compared the trends of changes in metabolites within subgroups of clinical patients who ultimately survived (SS) or did not survive (SNS), and how they related with those in endotoxemia. In total, there were 78 differential metabolites between SS and SNS groups at either t_0_ or t_24_. Among these, 23 were also differential for the endotoxemia group at t_6_. The direction and magnitude of changes in these 23 metabolites are shown in Table 3. When comparing the number of differential metabolites at either t_0_ and t_24_ of the SS and SNS groups, it is clear that the difference in metabolites becomes substantially more pronounced with time. Alignment of trends in the SS and SNS groups at t_24_ with those in endotoxemia at t_6_ revealed that the peak response to LPS is in line with the sepsis survivor metabolic response, especially at the first day into their treatment. In our previous metabolomics study [25], we identified the six hour time point as the peak of metabolic response in the endotoxemia model, stating that this time point represents the point of transition from the development and recovery phases of the response. The fact that the metabolic changes in the recovering patients shift towards this response pattern strengthens the notion that the metabolic, as well as transcriptional responses, characteristic to the endotoxemia model represent necessary and ‘healthy’ responses to an infectious stimuli. This is further evidence of likely allostatic response for survivors, that is, placing the host at the appropriate level of distress required for graceful resolution parallel to the one developed in the LPS model, versus the systemic maladaptation observed in non-survivors [28]. Based on this rationale, the endotoxemia model could be classified as a model of ‘normal, healthy responses.’ It is interesting to note that Matzinger [38] more than a decade ago proposed that the Toll-like receptors, including TLR4, evolved to serve as host defense mechanisms against major injury and trauma. Matzinger also proposed that the bacteria evolved to use this receptor system to its own advantage. This idea begins to explain why, when sufficiently controlled, LPS-induced responses might be protective and necessary rather than harmful.

**Table 3:** **The subset of metabolites having significantly different concentrations between SS and SNS groups at either clinical time points**

		**Sepsis survivors (number = 90)**		**Sepsis non-survivors (number = 31)**		**LPS (number = 15)**
**Metabolite name**	**Super pathway**	**t0**	**t24**	**t0**	**t24**	**t6**
2-hydroxybutyrate (AHB)	Amino acid	▲	-	▲	-	▲
N-acetylglycine	Amino acid	▽	▽	▲	▽	▲
xylose	Carbohydrate	-	▽	-	▲	▲
malate	Energy	-	▲	-	▲	▲
10-nonadecenoate (19:1n9)	Lipid	-	▲	-	▲	▲
2-hydroxypalmitate	Lipid	▲	-	▲	-	▲
hexanoylcarnitine (C6)	Lipid	▲	▲	▲▲	▲▲	▲
pregn steroid monosulfate	Lipid	▲	-	▽	-	▲
3-hydroxybutyrate (BHBA)	Lipid	-	▲	-	▲▲	▲▲
2-methylbutyroylcarnitine (C5)	Amino acid	▽	▽	▲	▲	▽
3-indoxyl sulfate	Amino acid	-	▽	-	▲	▽
5-oxoproline	Amino acid	-	▽	-	▲	▽
histidine	Amino acid	-	▽	-	▽	▽
isobutyrylcarnitine (C4)	Am ino acid	-	▽	-	▲	▽
N-acetylornithine	Amino acid	-	▽	-	▽▽	▽
tryptophan	Amino acid	-	▽	-	▽	▽
threitol	Carbohydrate	-	▽	-	▲	▽
phosphate	Energy	▽	▽	▲	▲	▽
1-linoleoyl-GPC (18:2)	Lipid	-	▽	-	▽	▽
1-oleoyl-GPC (18:1)	Lipid	-	▽	-	▽	▽
2-palmitoyl-GPC (16:0)	Lipid	▽	▽	▽▽	▽▽	▽
propionylcarnitine (C3)	Lipid	▲	▽	▲	▲	▽
allantoin	Nucleotide	▽	▽	▲	▲	▽

Changes from the healthy baseline, t_0,LPS_: ▲/▽: less than two fold change; ▲▲/▽▽: more than two fold change, −: there was not a significant difference between SS or SNS groups). LPS, lipopolysaccharide; SNS, sepsis non-survivors; SS, sepsis survivors.

The major goal of the CAPSOD study was to identify metabolite changes at sepsis presentation that predicted survival or death. Upon stratification of sepsis patients based on 28-day survival, the direction of change of 21 of 23 metabolites was the same in endotoxemia and sepsis survival (Table 3). The most important metabolite group that differentiated surviving and non-surviving CAPSOD patients was acyl-carnitines [28]. In our analysis, we observed a similar trend with all significantly changed acyl-carnitines exclusively higher than the t_0,LPS_ baseline at both time points in sepsis non-survivors (Additional file 4: Table S3), whereas for the surviving patients, around half of the acyl-carnitines were below the baseline. For the endotoxemia group, the direction of change in acyl-carnitine concentrations at t_6,LPS_ was the same as that of sepsis survivors (4 of total 12 acyl-carnitines were significant at t_6,LPS_).

Finally, a number of confounding factors need to be acknowledged. Firstly, the timing of the data collection, and, therefore, the phase of the response that is being studied, can vary greatly depending on the lag time from the initiating event to the presentation to an emergency department. Secondly, the nutritional input, being non-controlled either before or after the hospital admission, could have affected the plasma metabolite concentrations as an independent factor. Thirdly, some of the CAPSOD patients either had prior comorbidities that were likely to affect the metabolome, such as diabetes mellitus, or were also developing conditions which further exacerbated the response, including compromised renal function, a likely major contributor to the observed metabolome.

## Conclusions

Therapeutic strategies that are successfully translated into the clinic are very few and mostly non-specific in the field of critical care. This is due, in part, to the complex and dynamic physiological processes involved. Heterogeneity of the patient populations and consequent challenges in performing insightful clinical studies also have contributed to the lack of progress in this realm of medicine [3,39]. Emerging *-omics* tools that are capable of examining physiologic responses at the systems level are promising, especially for complex conditions, such as sepsis and SIRS [40]. The major caveat related to these tools is that since the biological processes are analyzed at a higher level, inter-species differences become as relevant to the response as the sought-after question itself. Therefore, utility of the animal models has been questioned recently in the scientific community [37,41].

The human endotoxemia model has been serving as a useful experimental platform for gaining insight into the mechanisms governing systemic inflammation. It is a recognized fact that this model does not fully replicate the magnitude of physiologic stress created by trauma or infection [13,14]; however, it gives researchers the opportunity to study the mechanisms underlying the response to systemic inflammation and relevant therapy options without the inter-species differences obscuring the interpretation of the results.

Progression of response to systemic inflammation induced by endotoxemia in immune cells has been described at the genomic level [16,30]. Moreover, comparison of the response to experimental stimuli and traumatic/infectious insults revealed significant overlap of common features both at the gene [15] and protein expression levels [34]. In the light of these observations, the current study aimed at an objective evaluation of the concordance between experimental and clinical cases of systemic inflammation and benchmarked endotoxemia against sepsis of various origins at the level of metabolic response. The plasma metabolome can be thought of as the metabolic fingerprint representative of the state of the body at any given time and provide information on the dominant regulatory mechanisms at various levels of cellular processes including transcription, translation and signal transduction. For effective provision of critical care, understanding the alterations in the plasma metabolome is crucial, because metabolite levels impact the regulation of anti-inflammatory defenses, in turn, through steering critical cellular processes and immune mechanisms. Therefore, we think that the assessment of the relevance of endotoxemia as an experimental model representing critical illness is important.

We believe that the observed concordance between the responses of LPS-treated subjects and sepsis patients at the metabolome level, despite observed variability in clinical data, strengthens the relevance of endotoxemia to clinical research as an elementary tool and gives valuable insights into the metabolic changes necessary for proper response to inflammatory stress at the systemic level.

## Key messages

- We compared the metabolic response at the peak of LPS-induced acute inflammation with those from sepsis patients
- For the metabolites shown to change significantly from the baseline, the direction and magnitude of the changes were in agreement with what was observed in sepsis patients.
- The metabolic response in SIRS patients was shown be distinct from those in endotoxemia or sepsis.
- Metabolic changes in the surviving sepsis patients shifted towards those observed in endotoxemia as their recovery proceeded.
- These observations strengthened the relevance of endotoxemia to clinical research as a valuable experimental tool which can enable further research on the development and assessment of rational clinical therapies to prevent sepsis mortality.

## Additional files

Additional file 1: Table S1. Number of outliers removed from the data before any statistical analysis. Additional file 2: Figure S1. Comparison of the variances of significant metabolites in the clinical groups with respect to those in the baseline (t_0,LPS_). Additional file 3: Table S2. Full list of the metabolites, their significance in each condition, direction and magnitude of the changes relative to the baseline (t_0,LPS_). Additional file 4: Table S3. Results of t-test for acyl-GPCs and acyl-carnitines, between SS and SNS groups at each time point, and between t_0,LPS_ and t_6,LPS_ in endotoxemia group, together with their direction of change from the common baseline t_0,LPS_. (Changes from the healthy baseline, t_0,LPS_: ▲/▽: less than 2 fold change; ▲▲/▽▽: more than 2 fold change).

## Abbreviations

APACHE II: Acute Physiology and Chronic Health Evaluation II
CAPSOD: Community Acquired Pneumonia and Sepsis Outcome and Diagnostics study
LPS: lipopolysaccharide
MAD: median absolute deviation
MS: mass spectrometry
SIRS: systemic inflammatory response syndrome
SNS: sepsis non-survivors
SOFA: Sequential Organ Failure Assessment
SS: sepsis survivors
TLR4: Toll-like receptor 4
