# Supplementary material for: Human metabolic response to systemic inflammation: assessment of the concordance between experimental endotoxemia and clinical cases of sepsis/SIRS
Source: Crit Care. 2015 Mar 3;19(1):71. doi: 10.1186/s13054-015-0783-2 (PMC4383069; doi:10.1186/s13054-015-0783-2)
Supplement: Additional file 1: Table S1. — Number of outliers removed from the data before any statistical analysis. [file 13054_2015_783_MOESM1_ESM.docx]

**Additional file 1: Table S1:** Number of outliers removed from the data before any statistical analysis.

|  | **LPS** | | **Sepsis** | | **SIRS** | |
| --- | --- | --- | --- | --- | --- | --- |
|  | **t0** | **t6** | **t0** | **t24** | **t0** | **t24** |
| **Outliers** | 117 | 118 | 1836 | 1476 | 469 | 348 |
| **Data points in total** | 2655 | 2655 | 21417 | 18939 | 5133 | 4425 |
| **% removed** | 4.4 | 4.5 | 8.5 | 7.8 | 9.1 | 7.9 |
