# Supplementary material for: Human metabolic response to systemic inflammation: assessment of the concordance between experimental endotoxemia and clinical cases of sepsis/SIRS
Source: Crit Care. 2015 Mar 3;19(1):71. doi: 10.1186/s13054-015-0783-2 (PMC4383069; doi:10.1186/s13054-015-0783-2)
Supplement: Additional file 2: Figure S1. — Comparison of the variances of significant metabolites in the clinical groups with respect to those in the baseline (t0,LPS). [file 13054_2015_783_MOESM2_ESM.docx]

**Additional file 2: Figure S1:** Comparison of the variances of significant metabolites in the clinical groups with respect to those in the baseline (t_0,LPS_).

**
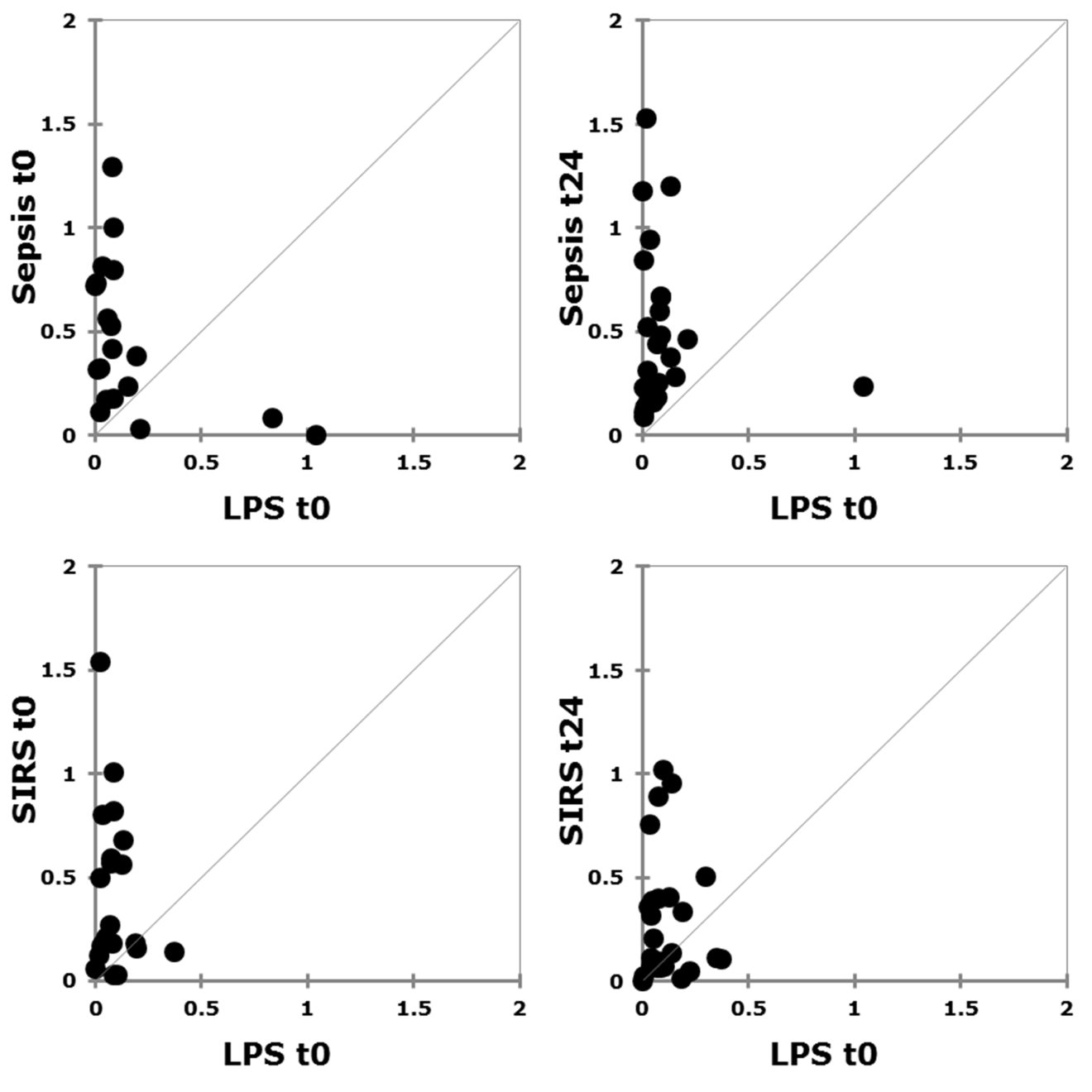
**
