# Supplementary material for: Human metabolic response to systemic inflammation: assessment of the concordance between experimental endotoxemia and clinical cases of sepsis/SIRS
Source: Crit Care. 2015 Mar 3;19(1):71. doi: 10.1186/s13054-015-0783-2 (PMC4383069; doi:10.1186/s13054-015-0783-2)
Supplement: Additional file 4: Table S3. — Results of t-test for acyl-GPCs and acyl-carnitines, between SS and SNS groups at each time point, and between t0,LPS and t6,LPS in endotoxemia group, together with their direction of change from the common baseline t0,LPS. (Changes from the healthy baseline, t0,LPS: ▲/▽: less than 2 fold change; ▲▲/▽▽: more than 2 fold change). [file 13054_2015_783_MOESM4_ESM.docx]

**Additional file 4: Table S3**: Results of t-test for acyl-GPCs and acyl-carnitines, between SS and SNS groups at each time point, and between t_0,LPS_ and t_6,LPS_ in endotoxemia group, together with their direction of change from the common baseline t_0,LPS_. (Changes from the healthy baseline, t_0,LPS_: ▲/▽: less than 2 fold change; ▲▲/▽▽: more than 2 fold change).

|  | **Significance of metabolites** | | | **Direction of change from common baseline** | | | | |
| --- | --- | --- | --- | --- | --- | --- | --- | --- |
|  | **Metabolites significantly differ between SS and SNS at** | | **LPS at** | **SS** | | **SNS** | | **LPS** |
|  |  |  |  |  |  |  |  |  |
|  | **t0** | **t24** | **t6** | **t0** | **t24** | **t0** | **t24** | **t6** |
| 1-arachidonyl-GPC (20:4) | No | Yes | No | - | ▽ | - | ▽▽ | - |
| 1-eicosatrienoyl-GPC (20:3) | Yes | Yes | No | ▽▽ | ▽ | ▽▽ | ▽▽ | - |
| 1-linoleoyl-GPC (18:2) | No | Yes | Yes | - | ▽ | - | ▽ | ▽ |
| 1-oleoyl-GPC (18:1) | No | Yes | Yes | - | ▽ | - | ▽ | ▽ |
| 1-palmitoleoyl-GPC (16:1) | Yes | Yes | No | ▽ | ▽ | ▽▽ | ▽▽ | - |
| 1-palmitoyl-GPC (16:0) | No | Yes | No | - | ▲ | - | ▽ | - |
| 1-stearoyl-GPC (18:0) | No | Yes | No | - | ▽ | - | ▽▽ | - |
| 2-palmitoyl-GPC (16:0) | Yes | Yes | Yes | ▽ | ▽ | ▽▽ | ▽▽ | ▽ |
| 2-methylbutyroylcarnitine (C5) | Yes | Yes | Yes | ▽ | ▽ | ▲ | ▲ | ▽ |
| isobutyrylcarnitine (C4) | No | Yes | Yes | - | ▽ | - | ▲ | ▽ |
| isovalerylcarnitine (C5) | No | No | No | - | - | - | - | - |
| tiglyl carnitine (C5) | Yes | Yes | No | ▽ | ▽▽ | ▲ | ▲ | - |
| deoxycarnitine | No | Yes | No | - | ▽ | - | ▲ | - |
| hexanoylcarnitine (C6) | Yes | Yes | Yes | ▲ | ▲ | ▲▲ | ▲▲ | ▲ |
| octanoylcarnitine (C8) | Yes | Yes | No | ▲ | ▲ | ▲▲ | ▲▲ | - |
| propionylcarnitine (C3) | Yes | Yes | Yes | ▲ | ▽ | ▲ | ▲ | ▽ |
| acetylcarnitine (C2) | Yes | Yes | No | ▽ | ▽ | ▲ | ▲ | - |
| butyrylcarnitine (C4) | Yes | Yes | No | ▽ | ▽ | ▲ | ▲ | - |
| decanoylcarnitine (C10) | Yes | Yes | No | ▲ | ▲ | ▲▲ | ▲▲ | - |
| cis-4-decenoyl carnitine | Yes | Yes | No | ▲ | ▲ | ▲ | ▲▲ | - |
